# BANA-Positive Plaque Samples Are Associated with Oral Hygiene Practices and Not CD4+ T Cell Counts in HIV-Positive Patients

**DOI:** 10.1155/2012/157641

**Published:** 2012-11-01

**Authors:** Cathy Nisha John, Lawrence Xavier Graham Stephen, Charlene Wilma Joyce Africa

**Affiliations:** ^1^Department of Periodontics and Oral Medicine, University of the Western Cape, Bellvile 7535, South Africa; ^2^Anaerobe Group, Department of Medical Biosciences, University of the Western Cape, Modderdam Road, Bellville 7535, South Africa

## Abstract

*Background*. The “red complex” microorganisms, namely, *Porphyromonas gingivalis*, *Treponema denticola*, and *Tannerella forsythia* are considered as potential pathogens causing HIV-associated periodontal diseases. Moreover, it has been recognized that an association exists between CD4+ T cell counts and periodontal disease progression. *Objective*. To establish whether CD4+ T cell counts or oral hygiene plays a greater role in producing BANA-positive results in HIV-associated periodontal disease. *Materials and Methods*. One hundred and twenty HIV-positive patients participated in the study, and their CD4+ T cell counts were obtained from their medical records. The six Ramfjord teeth were used for evaluating periodontal clinical indices and subgingival plaque sampling. BANA test was used for the detection and prevalence of the “red complex” bacteria in plaque samples. *Results*. A majority of 69.17% HIV-positive patients were BANA-positive. No significant associations were found between BANA and CD4+ T cell counts. A highly significant association was found between BANA with probing depth and clinical attachment level (*P* ≤ 0.0001) and between BANA and the use of interdental aids (*P* = 0.0168). *Conclusion*. HIV-associated periodontal diseases are strongly related to oral hygiene practices rather than the effect of CD4+ T cell counts, and the use of interdental aids was marked as a significant predictor of BANA-negative plaque samples.

## 1. Introduction 

Progressive human immunodeficiency virus infection (HIV) results in loss of immune response, especially those coordinated by CD4+ T lymphocyte cells [1]. Depletion of CD4 cell count renders the host vulnerable to opportunistic infections of the mouth [2]. A decrease in CD4+ T cell count as well as inadequate oral hygiene practices results in BANA-positive plaque accumulation and ultimately periodontal inflammation with destruction of the supporting tooth structures. The gradual increase in the proportions of gram-negative, anaerobic periodontopathogens, and their toxins can be released into the blood stream, thus activating the immune system [3, 4]. Inadequate oral hygiene can be considered as a risk indicator and/or risk predictor in the development of periodontal manifestations [5] which may be exacerbated by HIV infection.

The “red complex” microorganisms, namely, the potent periodontopathogenic bacteria *Porphyromonas gingivalis*, *Tannerella forsythia,* and *Treponema denticola* are considered as risk factors in HIV patients [6]. A rapid and simple chair-side bacteriological enzymatic test, BANA (N-benzoyl-DL-arginine-2-naphthylamide), could be reliably used in everyday practice to identify the presence or prevalence of “red complex” microorganisms [7]. *Porphyromonas gingivalis*, *Tannerella forsythia*, and *Treponema denticola* are cultivable BANA-positive plaque species which possess an enzyme capable of hydrolyzing the synthetic peptide of the BANA reagent [8]. BANA has shown to correlate well with the depth of the periodontal pocket and clinical indices which are used to diagnose periodontal disease [9]. The synergistic mechanisms between the “red complex” species result in an immune-inflammatory response [10]. 

In clinical periodontal practice, the BANA test has proved to be efficient in the diagnosis of periodontal disease and also in the bacteriologic monitoring of periodontally involved patients during the different phases of periodontal treatment [11]. The test provides equal accuracy in detecting these “red complex” species comparable to that obtained with DNA probes and immunology [7].

Microbial complexes of HIV-associated periodontal diseases are reported to be similar to those in HIV-negative individuals with periodontal diseases [12, 13]. No overt pathogen has been implicated for periodontal disease in HIV-seropositive individuals [14], although certain specific microbial complexes not commonly found in HIV-negative individuals may be responsible for chronic periodontal diseases in HIV-positive patients [15]. Studies conducted by Brady et al. [16], based on selective media, culture methods, and microscopy, did not demonstrate any characteristic difference in the microflora of HIV-infected and non-infected individuals with or without periodontal disease. The interaction of the “red complex” members with the epithelial cells in the subgingival environment leads to the destruction of host tissues.

The aim of the present study was to establish whether immunosuppression determined by the level of the CD4+ T cell counts in HIV-positive patients, or their oral hygiene practices play a greater role in the colonization of BANA-positive microorganisms such as *Porphyromonas gingivalis*, *Treponema denticola,* and *Tannerella forsythia, *implicated in the aetiology of HIV-associated periodontitis. 

## 2. Materials and Methods

### 2.1. Study Population

A cohort descriptive study was conducted involving a randomized selection of 120 male and female HIV-positive patients attending the infectious diseases clinic at Tygerberg Medical Hospital, South Africa. HIV-positive patients with various CD4+ T cell counts irrespective of their antiretroviral therapy were included in the study. Exclusion criteria included pregnancy, tuberculosis, diabetes, cardiovascular disease, autoimmune diseases, and also patients who were on antibiotic treatment and those who had undergone dental treatment 3 months prior to the study. Ethical committee clearance from the University of the Western Cape and written informed consent were obtained from all the participants prior to the start of the study. Information regarding demographic features, general health, HIV infection history, and most recent CD4+ T cell counts (usually within the last 3 months) were obtained from the patients' medical records. Viral loads were not recorded since the information was not always available. Factors predisposing to periodontal disease and oral hygiene habits were obtained from questionnaires and included questions pertaining to frequency of visits to the dentist (never, once a year, twice a year), frequency of tooth brushing (how many times a day), and the use of interdental aids (floss, interdental brushes, toothpicks).

### 2.2. Periodontal Procedures

Periodontal clinical measurements such as plaque index (PI), gingival index (GI), probing depth (PD), and clinical attachment level (CAL) were taken at the mesial aspect of six Ramfjord teeth. These measurements were performed by a single calibrated examiner using Williams periodontal probe. Intraexaminer Kappa index and intraclass coefficient correlation of agreement for periodontal pocket depth measurements ranged from 0.765 to 0.985. No oral hygiene instructions were given prior to the clinical examination.

### 2.3. Enzyme Assay

The BANA test was done as described by Loesche et al. [8]. The BANA reagent strip consisted of a plastic strip to which upper and lower reagent matrices were attached. After the removal of supragingival plaque, the subgingival plaque was collected from the site with the maximum pocket depth using a Gracey curette and dispersed on the lower reagent matrix of the BANA test strip. After moistening the upper matrix, the strip was folded to meet the upper and lower reagents and was incubated for 15 minutes at 55°C. If BANA-positive species were present when the test strip was opened, a permanent blue colour was obtained on the upper matrix.

### 2.4. Statistical Analysis

Data was analyzed using statistical programmes such as SAS (SAS Institute Inc., Cary, NC, USA). The Wilcoxon rank sum test was used to determine the association between CD4+ T cell counts and each of the clinical periodontal parameters with BANA. Pearson's Chi-square test was applied to determine the association between “red complex” and periodontal status. Cochran-Mantel-Haenszel test (CMH test) was used to testthe relationship between a pair of variables adjusting for the effect of other variables. Logistic regression model was used to determine the association between BANA and oral hygiene adjusting for ungrouped CD4+ T cell counts. A significance level of <0.01 rather than the usual <0.05 level indicated a highly significant result, while results with a *P*  value between 0.01 and 0.05 were referred to as marginally significant.

## 3. Results

Of the 120 participants included in the study, 55 (45.83%) were males and 65 (54.17%) were females with a mean age of 33.25 years (range: 20–55). A majority of 83 (69.17%) HIV-positive patients were reported as BANA-positive and 37 (30.83%) were BANA-negative (Table 1).

The mean CD4+ T cell count levels were 299.07 cells/mm³ and 280.79 cells/mm³ for BANA-positive and BANA-negative patients, respectively. There was no significant association found between BANA with CD4+ T cell counts (Wilcoxon test, *P*  =  0.7075); however, highly significant associations were observed between BANA with probing depth and clinical attachment level, while marginal significances were observed between BANA with plaque and gingival indices (Table 1).

By comparing BANA results with CD4+ T cell counts grouped into <200, 200–500 and >500 cells/mm³, 30.12% of the <200 cells/mm³ group, 59.04% of the 200–500 cells/mm³ group and 10.84% of the >500 cells/mm³ group were BANA-positive. No significant associations were found between BANA with grouped CD4+ T cell counts (Table 2). The results revealed that the presence of BANA-positive microorganisms were not related to CD4+ T cell counts as a whole or CD4+ T cell counts grouped into <200, 200–500 or >500 cells/mm³.

Of 83 (69.17%) BANA-positive patients, 62 (74.70%) brushed once a day and about 21 (25.30%) brushed twice a day while among the 37 (30.83%) BANA-negative patients, 22 (59.46%) admitted brushing once a day and only 15 (40.54%) admitted brushing twice a day. There were no significant associations found between BANA and the frequency of brushing (Table 3). A marginally significant association was apparent when comparing positive BANA results with the use of interdental aids (Chi-square test, *P*  =  0.0168). A majority of 70 (84.34%) of BANA-positive patients never used interdental aids while 13 (15.66%) of them reported using interdental aids. Among the BANA-negative patients, 24 (64.86%) never used interdental aids and 13 (35.14%) used interdental aids.

HIV-positive patients who brushed their teeth once a day and those who did not use any interdental aids to clean their teeth showed a greater prevalence of “red complex” as detected by the BANA test.  Table 4 shows the association between BANA and oral hygiene practices such as brushing and the use of interdental aids adjusted for grouped as well as ungrouped CD4+ T cell counts. The findings showed significant associations between BANA and interdental aids but not with brushing frequency. This explains the fact that the use of additional oral cleaning methods was essential and merely brushing was not enough to obtain a disease-free mouth. 

Moreover, CMH tests failed to demonstrate significant relationships between BANA and grouped as well as ungrouped CD4+ T cell counts even when adjusted for oral hygiene practices (Table 5).

From the results it can be suggested that oral hygiene maintenance plays a greater role in the aetiology of periodontal disease in HIV-positive patients than the level of immunosuppression. However, one must take into account the fact that this study included HIV patients whether or not they were on therapy. This may have influenced the results obtained. 

## 4. Discussion

Studies have suggested an increase in the severity and prevalence of periodontal diseases in HIV-positive populations. The primary aetiology of periodontal diseases is the formation and adhesion of dental plaque on the surfaces of the tooth. Dental plaque involves a community of microorganisms found as a biofilm on the tooth surface and develops under different conditions and environments. In the periodontal disease progression, “red complex” is considered as the most significant cluster among the bacterial clusters [17], as the “red complex” pathogens increase in numbers and prevalence with increasing clinical parameters of the disease [18]. 

Since periodontal disease is a polymicrobial infection, a coaggregation of subgingival microorganisms exists in HIV-positive patients. Among them *Porphyromonas gingivalis*, *Treponema denticola*, and *Tannerella forsythia* can be considered as true pathogens in HIV progression. However, other studies reported a lower prevalence of these putative bacteria in HIV-positive compared with HIV-negative individuals [19, 20]. The clinical signs of HIV-associated periodontitis are always related to gingival inflammation, profound gingival bleeding, increased probing depth, and loss of clinical attachment and hence periodontal disease may be determined as one of the first clinical representations of an undiagnosed HIV infection [21]. 

In the present investigation, BANA test showed significant associations with all the periodontal indices. Clinical attachment level and probing depth were found highly significant (*P*  ≤  0.0001) and plaque (*P*  =  0.0248), and gingival (*P*  =  0.038) indices were found to be marginally significant with BANA. This investigation's results clearly indicate the relationship between BANA-positive species and their role in periodontal disease. A study by Puscasu et al. [22], on 61 adult patients with gingivitis or periodontitis, observed a statistical correlation with the severity of periodontal destruction and BANA but they failed to determine a statistical correlation between plaque index and BANA. However, their study used both supragingival and subgingival plaque deposits for BANA test, where supragingival plaque harbours less anaerobic BANA-positive species which could thus prevent their detection. On the contrary, in the present study, only subgingival plaque samples were taken to detect the “red complex,” where they colonize abundantly. This could account for their positive response to the BANA test.

It has been suggested that the level of immunosuppression or the decrease in the CD4+ T cell count determines the progression of periodontal disease. Highly severe immunosuppression in HIV patients may exacerbate preexisting periodontitis, considering HIV infection as a modifier of periodontal disease [23]. However, in the present investigation, BANA test was not found to be correlated with absolute CD4+ T cell counts nor with grouped CD4+ T cell counts even when adjusted for oral hygiene. The interesting finding was that an increase in the “red complex” microorganisms in HIV infections was not clearly demonstrated to be due to the decrease in CD4+ T cell counts, per se, but it could be due to the lack of oral hygiene maintenance that leads to the colonization of “red complex” pathogens. Most of the HIV patients examined brushed only once a day (70%) and never used interdental aids (78.33%) thus indicating less effort on the patient's part to maintain better oral hygiene, ultimately leading to gingival inflammation and periodontal diseases. Lack of proper oral hygiene may lead to the accumulation of periodontal microorganisms in the mouth resulting in a positive response to the BANA test. BANA was not significantly related to the frequency of brushing (Chi-square test, *P*  = 0.0925) but there was a marginally significant relationship between BANA and the use of interdental aids (Chi-square test, *P*  =  0.0168). Inadequate brushing leads to the accumulation of both supragingival and subgingival plaque making oral hygiene difficult. Moreover, it was evident that association of periodontal clinical parameters such as plaque and gingival indices, probing depth and loss of clinical attachment with BANA positivity in HIV patients suggests improper maintenance of oral hygiene. 

An alternate analysis using the logistic regression model gave virtually identical results. Not adjusting for CD4+ T cell counts, the use of interdental aids was significant with *P*  =  0.0168 (Chi-square test). Adjusting for CD4+ T cell counts in a logistic regression model, the *P*  value for interdental aids was still significant with *P*  =  0.0244. The odds ratio was 0.35 for interdental aids, and the 95% confidence interval was 0.14 to 0.85.

Even when only a minority of the study population used interdental aids as an additional cleaning device, it marked a major difference in the oral hygiene maintenance compared to the frequency of brushing. As one would expect, more frequent brushing and interdental use tends to provide better plaque control and improved gingival health. Clinicians must motivate the patients and highlight the benefits of additional cleaning devices to clean their teeth. Overall, the oral hygiene status among the HIV-positive patients who participated in the study was poor which may explain the presence of BANA-positive pathogens.

The immunosuppression of HIV patients influences the prevalence of periodontal diseases which in turn is exacerbated by their poor oral hygiene. Since the current investigation was strictly restricted for the detection of “red complex” subgingival pathogens only, knowledge of any relation with one or a combination of other periodontopathic bacteria causing destruction of the periodontium cannot be ignored. This could be considered as a limitation of the study since other bacterial species that might have been present subgingivally were not detected. The present study included patients who had not received any form of dental treatment 3 months prior to the study, thereby eliminating the bias of those visiting dental clinics for oral complaints. Some of the HIV patients in the study were on antiretroviral therapy, which could explain the reason of not finding any association between BANA-positive pathogens and their CD4+ T cell count levels. Earlier intervention to antiretroviral therapies and the widespread use of antiretroviral therapy could limit the exposure to immunosuppression in HIV patients. The regular use of antimicrobials in the form of antiretroviral therapy may reduce the virulence of the subgingival microbiota in HIV-positive patients, even under severe immunosuppression.

This study employed the Ramfjord teeth instead of a full-mouth assessment for the periodontal status of the study group. While some researchers have demonstrated the epidemiological validity of the Ramfjord teeth in representing the periodontal status of the whole mouth [24–29], others have reported the introduction of a bias (either positively or negatively), thus making it unsuitable for determining periodontal disease severity or prevalence [30–32]. We elected to use the Ramfjord teeth since it was found to reduce time, cost, patient, and examiner fatigue, while also providing a practical alternative to the 168 measurements for each clinical parameter required to characterise the prevalence and severity of periodontal disease in a single whole mouth [28]. However, we are mindful of the limitations of partial mouth measurements. Measurement of sites on the buccal side of the tooth have been reported to show better reliability than measurement on the lingual side because of better visibility to the examiner. A better representation may therefore be achieved by measuring several sites on a tooth instead of just one aspect as we have done. Furthermore, partial recording techniques were shown to reduce the severity of periodontal destruction scores to almost half of that demonstrated in full mouth examinations, resulting in an underestimation of both the extent and prevalence of periodontal disease in some studies [28, 33]. 

## 5. Conclusion

The presence of “red complex” pathogens (as demonstrated by the BANA test) was not associated with the level of immunosuppression nor CD4+ Tcell counts in HIV-positive patients. Although immunosuppression plays a major role in the periodontal inflammation, improper oral hygiene may aggravate its severity and progression. The “red complex” subgingival pathogens were closely related to the clinical parameters of inflammation and periodontal destruction and appeared meaningful in periodontal diagnosis. The results reported in this investigation would imply that an association existed between the presence of BANA-positive plaques and the use of interdental aids, and virtually no evidence of an association with CD4+ T cell counts. The high prevalence of periodontal manifestations underlines the need for routine dental visits and treatment with a proper maintenance of oral hygiene. A greater emphasis on oral health and prevention of periodontal disease is necessary especially among HIV-infected individuals. 

## Figures and Tables

**Table 1:** CD4+ T cell counts and clinical indices relative to frequency of BANA.

Variables	BANA						*P* value
	Positive (*N* = 83)			Negative (*N* = 37)			
	Mean (SD)	Median	Minimum–Maximum	Mean (SD)	Median	Minimum–Maximum	
CD4+ T cell counts^a^	299.07 (157.16)	300	61–859	280.79 (137.6)	256	36–562	0.7075
Plaque index^b^	2.64 (0.46)	2.9	1.3–3	2.35 (0.64)	2.5	0.8–3	0.0248
Gingival index^c^	2.83 (0.35)	3	1.5–3	2.59 (0.59)	2.9	0.5–3	0.0348
Probing depth^d^	5.06 (1.02)	5	2.9–6.8	4.12 (0.76)	4	2.9–5.8	<0.0001
Clinical attachment level^e^	5.6 (1.1)	5.9	3–7.3	4.59 (0.83)	4.4	3–6.1	<0.0001

^a^No statistical significance observed between CD4+ T cell counts and BANA test (Wilcoxon rank sum test).

^b,c^Marginally statistical significance observed between BANA and plaque and gingival indices (Wilcoxon rank sum test).

^d,e^Highly statistical significance observed between BANA and probing depth and clinical attachment level (Wilcoxon rank sum test).

**Table 2:** Grouped CD4+ T cell counts relative to BANA.

BANA	Grouped CD4+ T cell counts (*P* = 0.9989)			Total
	<200	200–500	>500	
Negative	11 (29.73%)	22 (59.46%)	4 (10.81%)	37 (30.83%)
Positive	25 (30.12%)	49 (59.04%)	9 (10.84%)	83 (69.17%)

*No statistical significance observed between BANA and grouped CD4+ T cell counts (Pearson's Chi-square test).

**Table 3:** BANA relative to oral hygiene practices.

BANA	Brushing (*P* = 0.0925)		Interdental aids (*P* = 0.0168)	
	Once a day	Twice a day	No	Yes
Negative (*n* = 37)	22 (59.46%)	15 (40.54%)	24 (64.86%)	13 (35.14%)
Positive (*n* = 83)	62 (74.70%)	21 (25.30%)	70 (84.34%)	13 (15.66%)

*No statistical significance observed between BANA positivity and brushing (Pearson's Chi-square test).

*Statistical significance observed between BANA positivity and interdental aids (Pearson's Chi-square test).

**Table 4:** Association between BANA with oral hygiene adjusted for grouped and ungrouped CD4+ T cell counts.

			Ungrouped CD4+ T cell counts		
Independent variables		Grouped CD4+ T cell counts (*P* values) (CMH test)	(Logistic regression model)		
			Odds ratio	95% CI	*P* values
BANA	Brushing^a^	0.0897	0.50	0.22–1.14	0.1154
	Interdental aids^b^	0.0174	0.35	0.14–0.85	0.0244

^a^No statistical significance observed between BANA and brushing with grouped and ungrouped CD4+ T cell counts.

^b^Statistical significance observed between BANA and interdental aids with grouped and ungrouped CD4+ T cell counts.

**Table 5:** Association between BANA with grouped and ungrouped CD4+ T cell counts adjusted for oral hygiene.

Independent variables		*P* values
BANA	Grouped CD4+ T cell counts	0.9423
	Ungrouped CD4+ T cell counts	0.9943

*No statistical significance observed between BANA and grouped and ungrouped CD4+ T cell counts (CMH test).
